# Health App Use Among US Mobile Phone Users: Analysis of Trends by Chronic Disease Status

**DOI:** 10.2196/mhealth.7832

**Published:** 2017-12-19

**Authors:** Rebecca Robbins, Paul Krebs, Ram Jagannathan, Girardin Jean-Louis, Dustin T Duncan

**Affiliations:** ^1^ Department of Population Health NYU School of Medicine New York, NY United States; ^2^ Emory Global Diabetes Research Center Hubert Department of Global Health Emory Rollins College of Public Health Atlanta, GA United States; ^3^ Center for Data Science New York University New York, NY United States

**Keywords:** smartphone, telemedicine, chronic disease

## Abstract

**Background:**

Mobile apps hold promise for serving as a lifestyle intervention in public health to promote wellness and attenuate chronic conditions, yet little is known about how individuals with chronic illness use or perceive mobile apps.

**Objective:**

The objective of this study was to explore behaviors and perceptions about mobile phone–based apps for health among individuals with chronic conditions.

**Methods:**

Data were collected from a national cross-sectional survey of 1604 mobile phone users in the United States that assessed mHealth use, beliefs, and preferences. This study examined health app use, reason for download, and perceived efficacy by chronic condition.

**Results:**

Among participants, having between 1 and 5 apps was reported by 38.9% (314/807) of respondents without a condition and by 6.6% (24/364) of respondents with hypertension. Use of health apps was reported 2 times or more per day by 21.3% (172/807) of respondents without a condition, 2.7% (10/364) with hypertension, 13.1% (26/198) with obesity, 12.3% (20/163) with diabetes, 12.0% (32/267) with depression, and 16.6% (53/319) with high cholesterol. Results of the logistic regression did not indicate a significant difference in health app download between individuals with and without chronic conditions (*P*>.05). Compared with individuals with poor health, health app download was more likely among those with self-reported *very good health* (odds ratio [OR] 3.80, 95% CI 2.38-6.09, *P*<.001) and *excellent health* (OR 4.77, 95% CI 2.70-8.42, *P*<.001). Similarly, compared with individuals who report *never or rarely* engaging in physical activity, health app download was more likely among those who report exercise 1 day per week (OR 2.47, 95% CI 1.6-3.83, *P*<.001), 2 days per week (OR 4.77, 95% CI 3.27-6.94, *P*<.001), 3 to 4 days per week (OR 5.00, 95% CI 3.52-7.10, *P*<.001), and 5 to 7 days per week (OR 4.64, 95% CI 3.11-6.92, *P*<.001). All logistic regression results controlled for age, sex, and race or ethnicity.

**Conclusions:**

Results from this study suggest that individuals with poor self-reported health and low rates of physical activity, arguably those who stand to benefit most from health apps, were least likely to report download and use these health tools.

## Introduction

Health conditions, such as hypertension and obesity, are associated with lower quality of life and increased health care costs [1]. Individuals with chronic conditions are often burdened by complex treatment regimens [2-4], and poor adherence to treatment in chronically ill populations is common [3]. Evidence suggests that chronic illness is exacerbated by modifiable health and lifestyle factors such as sedentary behavior and unhealthy dietary habits [5-8]. Mobile technologies have been tailored to chronically ill populations [9,10], but little work has examined current preferences, attitudes, and use of mobile health in these groups.

These behavioral factors pose a significant challenge for effective chronic disease management. For instance, medication nonadherence is prevalent in populations with chronic conditions and is associated with increased risk for hospitalization and mortality [8]. Other modifiable factors, such as poor stress management, have also been linked to increased mortality risk in patients with chronic conditions [7]. Unfortunately, poor adherence to disease management increases risk for additional chronic diagnoses and is associated with higher health care costs. Specifically, per person health care costs increase from US $211 in patients with a single chronic disease to US $13,000 in patients with two or more chronic conditions [4]. Identifying methods for optimal disease management and health promotion among chronic disease populations is critical, and mobile health technologies may aid in these efforts.

Overall, mobile technology is increasingly prolific across the populations. Approximately two-thirds of adults in the United States own a mobile phone [10]. Mobile phones feature robust capacities such as Bluetooth, location sensing, and software apps [11]. These technologies can help users perform a variety of tasks such as tracking exercise and providing reminders to take a medication or to go for a walk [12,13]. A vast number of health-related apps are available to consumers; over 100,000 apps are currently available for assisting users in achieving diverse objectives, from quitting smoking to taking more steps [13]. A number of apps have been designed specifically for populations with chronic conditions. A review study found over 3500 apps designed for chronic conditions, with a majority of available apps tailored to assist patients with diabetes or depression [14].

Health apps are a promising future direction for chronic disease treatment and care [11,15,16]. For instance, mobile technologies have capabilities to nudge modifiable actions, such as medication adherence or making healthy lifestyle choices, and thus offer promise for assisting with treatment and care among individuals with chronic conditions [17,18]. The Centers for Disease Control and Prevention Healthy People 2020 goals include a call for technology to improve population health and disease management [19]. Despite the potential for health apps in management or treatment of chronic conditions, we know little about current health app use among individuals with these conditions. Research to date has examined the use and effects of health apps [20] as well as beliefs about health apps among healthy populations [21,22]. We know little, however, about the beliefs individuals with chronic conditions hold regarding health apps, making this study novel in its approach.

The aim of this study was to examine beliefs related to perceived efficacy of health apps and current health app behaviors among individuals with and without chronic conditions in a national sample of mobile phone users. We utilized data collected in a previous study [20] among mobile phone and mobile health use broadly to answer this question. Results of this study on behaviors regarding health apps among individuals with and without chronic conditions outline a direction for future research and design on apps for chronic disease patients that are tailored to their unique behavior patterns among individuals with particular chronic conditions.

## Methods

### Sample and Procedures

This study utilizes data from a national cross-sectional sample of mobile phone users in the United States [20]. Data were collected in 2015 from a sample of 1604 adult respondents. Owning a mobile phone was an inclusion criterion for this study. To provide a national sample efficiently, Toluna, a survey management company, was contracted to deliver the survey. Toluna identified participants by emailing their existing panel of participants and then employing quota sampling to gather data from groups who are traditionally underrepresented in technology-related surveys. Sampling quotas set before data collection were as follows: 50% male and 50% female; 50% with high school or higher level of education; 60% earning less than US $50,000 and 40% earning more than US $50,000; and 30% white, 30% Latino or Hispanic, 30% black, and 10% Asian.

Items on health apps were developed for this study following standard item design techniques [23]. First, research assistants conducted Web-based queries to identify health app uses using search terms such as *uses* and *capabilities*. This generated a long list of potential health app uses and functions for these apps among users. Next, we met and devised a list of health apps they encountered in their social networks, or in their research or experience interacting with patients. Then, we organized responses thematically and deleted redundant inputs. The final list of items for this study, such as reasons for downloading or perceived efficacy of health apps, was identified and incorporated in this study.

The survey was pilot-tested with a sample of nonresearch team members using cognitive interviewing techniques to ensure the survey was clear and the items were easy to understand. Before taking the online questionnaire, participants provided their consent to participate in the study. The survey took on average 9 min to complete. This study was approved by the New York University School of Medicine Institutional Review Board (IRB #i14-02046). As this research included surveys with human subjects, participants’ consent for participation was obtained before any data capture activities. A copy of the consent form may be provided upon request. Data are retained by the corresponding author. Any individuals interested in obtaining a copy of the dataset will be addressed promptly.

### Measures

The survey consisted of 36 questions, assessing demographics (age, gender, race, income, and education), health (chronic condition diagnoses, self-rated health, and physical activity), reasons for downloading and not downloading health apps, frequency of using health apps, and perceived efficacy of health apps.

Participants were first asked “Have you ever downloaded an ‘app’ to track anything relating to your health?” Participants who reported health app download were prompted with several follow-up questions about reasons for download (eg, “To track what I eat” and “Help with weight loss”). Overall use of health app use was measured by asking participants how frequently they use health apps, both frequency of each session (response options ranged from “less than once a month” to “2 or more times per day”) and duration of each session (response options ranged from “1-10 minutes” to “more than 30 minutes”). Participants who reported using a health app were asked to report perceived efficacy of health apps (on a scale from “made my health worse” to “very much improved my health”). Chronic condition diagnoses were collected via self-report. Chronic illnesses were selected if prevalence was at least 5% in this sample (eg, hypertension, obesity, diabetes, depression, and high blood cholesterol). Chronic conditions comprising less than 5% on the sample included cancer (n=64, 4%), chronic obstructive pulmonary disease (n=62, 4%), heart attack (n=51, 3%), stroke (n=51, 3%), substance abuse (n=45, 3%), ulcers (n=38, 3%), liver disease (n=17, 1%), and human immunodeficiency virus (n=10, 1%).

### Statistical Methods

Differences in response between the conditions (no chronic condition, hypertension, obesity, diabetes, depression, and high blood cholesterol) were examined by demographic factors (age, race, education, sex, self-rated health, and physical activity). As discussed, chronic illnesses were selected if prevalence was at least 5% in the sample (eg, hypertension, obesity, diabetes, depression, and high blood cholesterol).

Differences in responses to frequency of health app use, perceived app efficacy, and reasons for download were also examined by condition. Finally, logistic regression was performed utilizing the generalized linear model technique. Health app download was examined with chronic condition, self-reported health, and physical activity. In the case of health conditions, a variable was created that was coded to indicate condition (eg, no condition, hypertension, obesity, diabetes, depression, and high cholesterol) to allow for analyses between conditions. Consistent with previous literature [24], age, sex, and race or ethnicity were included in the models as covariates. We analyzed the data using SPSS version 22 (Armonk, New York).

## Results

### Sample Characteristics

A total of 7189 people visited the survey page, 6871 (95.61%) agreed to participate in the survey, 2089 (29.04%) completed the survey, and 485 (6.75%) were randomly removed because of overfilling of sociodemographic quotas. Table 1 displays demographic characteristics of the sample. Overall, 49.56% (795/1604) of the study sample was female, with a mean age of 40.1 (SD 15.8) years. Among the participants, 35.47% (569/1604) were white. Among participants, 37.59% (603/1604) reported *very good* to general health and 13.52% (217/1604) reported *excellent*. Regarding physical activity, the majority of individuals reported being active *3-4 days per week* (35.09%, 563/1604).

Of the 1604 individuals in the study, the most prevalent chronic conditions included hypertension (n=364, 22.69%), obesity (n=198, 12.34%), diabetes (n=163, 10.16%), depression (n=267, 16.64%), and high cholesterol (n=319, 19.89%).

### Mobile Health App Use, Frequency, and Perceived Efficacy by Chronic Condition

Table 2 displays differences by condition for number of health apps and frequency of use. Regarding the number of apps, *1-5 apps* was reported by 38.9% (314/807) of individuals with no conditions, 6.6% (24/364) with hypertension, 15.0% (34/163) with diabetes, 7.6% (16/198) with obesity, 25.8% (69/267) with depression, and 27.6% (88/319) with high cholesterol. In addition, reason for health app download varied significantly for *help me watch/improve what I eat* (*P*=.00), *weight loss* (*P*=.01), *track a health measure* (*P*=.04), and *help me relax* (*P*=.01) by chronic condition.

### Examining Mobile Health App Characteristics by Chronic Condition

Among individuals with no chronic conditions, 66.0% (533/807) reported health app download. Of the individuals with one chronic condition, 53.4% (189/352) reported health app download, whereas just less than half (47.0%, 211/449) of individuals with a chronic condition reported health app download. Significant differences in app download were found by condition (χ^2^_2_=44.3, *P*=.003) and examined using logistic regression models. See Table 3.

**Table 1:** Demographic characteristics of mobile phone users in the United States by chronic condition (N=1604).

Variable		No condition (n=807)	Hypertension (n=364)	Obesity (n=198)	Diabetes (n=163)	Depression (n=267)	High cholesterol (n=319)	*P* value
		n (%)	n (%)	n (%)	n (%)	n (%)	n (%)	
Age^a^		33.8 (12.8)	40.1 (15.8)	33.8 (16.5)	41.4 (16.5)	50.6 (16.1)	38.6 (15.9)	<.001
**Sex**								<.001
	Male	399 (49.4)	193 (53.0)	71 (35.8)	84 (51.5)	91 (34.1)	203 (63.6)	
	Female	408 (50.6)	170 (46.7)	125 (63.1)	79 (48.5)	176 (65.9)	116 (36.4)	
**Education**								<.001
	Less than 12th grade	43 (5.3)	17 (4.7)	9 (4.5)	7 (4.3)	20 (7.5)	12 (3.8)	
	High school or General Equivalency Degree	388 (48.1)	142 (39.0)	73 (36.8)	61 (37.4)	120 (44.9)	115 (36.1)	
	Some college	176 (21.8)	110 (30.2)	66 (33.3)	41 (25.2)	75 (28.1)	91 (28.5)	
	Bachelor’s degree	148 (18.3)	61 (16.7)	35 (17.6)	40 (24.5)	38 (14.2)	66 (20.7)	
	Graduate degree	52 (6.4)	34 (9.3)	15 (7.6)	14 (8.6)	14 (5.2)	35 (11.0)	
**Race or ethnicity**								<.001
	African American/black	219 (27.1)	111 (30.5)	58 (29.3)	43 (26.4)	61 (22.8)	54 (16.9)	
	Asian	70 (8.7)	15 (4.12)	10 (5.1)	10 (6.1)	9 (3.3)	15 (4.7)	
	White	199 (24.6)	175 (48.1)	77 (38.9)	65 (39.8)	125 (46.8)	173 (54.2)	
	Native American	6 (0.7)	2 (0.5)	2 (1.0)	1 (0.6)	4 (1.5)	3 (0.94)	
	Latino/Hispanic	279 (34.6)	56 (15.4)	48 (24.2)	44 (26.9)	62 (23.2)	65 (20.4)	
	Other	34 (4.2)	5 (1.4)	3 (1.5)	0 (0.0)	6 (2.3)	9 (2.8)	
**Self-reported health**								<.001
	Poor	9 (1.1)	5 (1.4)	1 (0.5)	7 (4.3)	21 (7.8)	5 (1.6)	
	Fair	44 (5.5)	17 (4.7)	7 (3.5)	46 (28.2)	79 (29.6)	17 (5.3)	
	Average	254 (31.5)	26 (7.1)	20 (10.1)	80 (49.1)	118 (44.2)	26 (8.2)	
	Very good	417 (51.7)	24 (6.6)	23 (11.6)	33 (20.3)	73 (27.3)	24 (7.5)	
	Excellent	155 (19.2)	7 (1.9)	6 (3.0)	17 (10.4)	28 (10.5)	7 (2.19)	
**Physical activity**								<.001
	Never	115 (14.3)	21 (5.8)	14 (7.1)	5 (3.1)	38 (14.2)	70 (21.9)	
	1 day	86 (10.6)	7 (1.9)	10 (5.1)	1 (0.)	26 (9.74)	38 (11.9)	
	2 days	198 (24.5)	17 (4.7)	22 (11. 1)	13 (7.9)	40 (15.0)	58 (18.1)	
	3-4 days	340 (42.1)	28 (7.7)	25 (12.6)	24 (14.7)	48 (18.0)	98 (30.7)	
	5-7 days	140 (17.3)	14 (3.8)	8 (4.0)	14 (8.6)	31 (11.6)	55 (17.2)	

^a^Represents mean and standard deviation.

**Table 2:** Responses to health app use, frequency, and perceived efficacy by chronic condition (N=1604).

Variable		No condition		Hypertension		Obesity		Diabetes		Depression		High cholesterol		*P* value
		n (%)		n (%)		n (%)		n (%)		n (%)		n (%)		
**Number of health apps**														<.001
	1-5 apps	314 (38.9)		24 (6.6)		34 (17.2)		16 (9.8)		69 (25.8)		88 (27.6)		
	6-10 apps	55(6.8)		2(0.5)		12 (6.1)		2 (1.0)		15 (5.6)		18 (5.6)		
	11-15 apps	52 (6.4)		2 (0.5)		1 (0.5)		2 (1.2)		6 (2.2)		4 (1.3)		
	16-20 apps	77 (9.5)		4 (1.1)		1 (0.5)		2 (1.2)		4 (1.5)		5 (1.6)		
**Frequency of health app use**														.001
	Less than once a month	32 (4.0)		0 (0.0)		3 (1.5)		0 (0.0)		15 (5.6)		9 (2.8)		
	A few times a month	34 (4.2)		6 (1.60)		4 (2.0)		0 (0.0)		8 (3.0)		16 (5.0)		
	A few times each week	119 (14.70)		7 (1.9)		10 (5.1)		5 (3.1)		21 (7.9)		33 (10.3)		
	About 1 time each day	211 (26.1)		10 (2.7)		11 (5.6)		5 (3.1)		28 (10.5)		34 (10.7)		
	2 or more times a day	172 (21.3)		10 (2.7)		26 (13.1)		20 (12.3)		32 (12.0)		53 (16.6)		
**Duration of health app use**														.17
	1-10 min	312 (38.7)		78 (21.4)		52 (26.3)		33 (20.2)		77 (28.8)		66 (20.7)		
	11-30 min	339 (42.0)		50 (13.7)		47 (23.7)		32 (19.6)		48 (18.0)		55 (17.2)		
	More than 30 min	72 (8.9)		27 (7.4)		20 (10.1)		20 (12.3)		25 (9.4)		24 (7.5)		
**Perceived efficacy of health apps**														.13
	Made my health worse	20 (2.5)		2 (0.5)		2 (1.0)		2 (1.2)		1 (0.4)		3 (0.9)		
	Did not help at all	51 (6.3)		15 (4.1)		15 (7.6)		14 (8.6)		9 (3.4)		6 (1.9)		
	Just a little improved	164 (20.3)		47 (12.9)		43 (21.7)		43 (26.4)		25 (9.4)		21 (6.6)		
	Somewhat	237 (29.4)		47 (12.9)		38 (19.2)		45 (27.6)		43 (16.1)		24 (7.5)		
	Very much improved	215 (26.6)		44(12.1)		47 (23.7)		46 (28.2)		41 (15.4)		31 (9.7)		
**Reason for health app download**														
	Track activity or exercise I get	370 (45.8)		97 (26.6)		77 (38.9)		53 (32.5)		82 (30.7)		92 (28.8)		.06
	Help me watch/improve what I eat	335 (41.5)		85 (23.4)		76 (38.4)		52 (31.9)		90 (33.7)		72 (22.6)		.00
	Weight loss	333 (41.3)		77 (21.2)		80 (40.4)		49 (30.1)		86 (32.2)		66 (20.7)		.01
	Track a health measure	189 (23.4)		60 (16.5)		38 (19.2)		42 (25.8)		50 (18.7)		54 (16.9)		.04
	Help me relax	143 (17.7)		3 (0.8)		8 (4.0)		6 (3.7)		17 (6.4)		33 (10.3)		.01

**Table 3:** Health app download, self-reported health, and physical activity by chronic condition (N=1604).

Variable		Unadjusted models		Adjusted models^a^	
		Odds ratio (95% CI)	*P* value	Odds ratio (95% CI)	*P* value
**Chronic conditions**					
	No chronic condition	Reference		Reference	
	Hypertension	0.34 (0.21-0.53)	<.001	0.74 (0.45-1.22)	.24
	Obesity	1.18 (0.72-1.94)	.51	1.63 (0.96-2.77)	.07
	Diabetes	0.61 (0.36-1.04)	.07	1.24 (0.69-2.24)	.47
	Depression	0.72 (0.52-1.00)	.05	0.91 (0.64-1.28)	.58
	High cholesterol	0.46 (0.35-0.59)	<.001	1.00 (0.73-1.37)	.99
**Self-reported health**					
	Poor health	Reference		Reference	
	Fair health	1.07 (0.69-1.66)	.76	1.30 (0.82-2.07)	.27
	Good health	1.29 (0.86-1.94)	.23	1.55 (1.00-2.40)	.05
	Very good health	3.28 (2.12-5.06)	.000	3.80 (2.38-6.09)	.000
	Excellent health	5.36 (3.14-9.14)	.000	4.77 (2.70-8.42)	.000
**Physical activity**					
	Never	Reference		Reference	
	1 day per week	3.08 (2.05-4.64)	.000	2.47 (1.60-3.83)	.000
	2 days per week	5.38 (3.78-7.66)	.000	4.77 (3.27-6.94)	.000
	3-4 days per week	6.15 (4.43-8.54)	.000	5.00 (3.52-7.10)	.000
	5-7 days per week	5.13 (3.53-7.45)	.000	4.64 (3.11-6.92)	.000

^a^Model adjusted for age, sex, race or ethnicity.

In unadjusted models, individuals who were less likely to report health app download included those with hypertension (*P*<.001), depression (*P*<.05), and high cholesterol (*P*<.001). No chronic conditions were significant predictors of health app download after adjusting for covariates (age, sex, and race or ethnicity). However, *very good* (*P*<.001) and *excellent* (*P*<.001) self-reported health were strong predictors of health app download in adjusted models and in models adjusting for covariates. These findings were consistent in adjusted models. Similarly, individuals reporting 1 day or more of physical activity were significantly more likely to report health app download compared with those who reported *never* for physical activity (*P*<.001).

## Discussion

### Principal Findings

Health apps and other mobile technologies hold promise as tools for health promotion among healthy individuals as well as those with chronic illness [13]. Over 3000 apps exist that are targeted to chronically ill populations [18]. Although the majority of research has examined effects of apps tailored to assist individuals with chronic illness [14], little attention has been paid to health app use between individuals with poor general health and/or chronic illness. Our study recruited a sample of mobile phone users throughout the United States, as well as a large proportion of minority participants to compare beliefs and attitudes, as well as use of health apps, between individuals with no chronic illness and those with specific diagnoses.

Previous research has examined motivation to download health apps among healthy populations, including college students and adults. In the study conducted by Kwon and colleagues, mobile health app use was associated with perceived efficacy of apps [21]. In two studies examining health app use among healthy adults, one found that health app use was associated with perceived usefulness of apps [22], and another study found that high perceived cost was a deterrent to health app download but health app use was overall quite high among the population [20]. The published literature on health app use and download has thus largely emphasized trends, use, and beliefs about health apps among general, and largely healthy, populations, with little attention to health app use between individuals with poor health and chronic illness.

Our study provides a meaningful contribution to the literature, in examining beliefs about health apps among those with good health indicators as well as poor health indicators. Our study found that approximately one-third of individuals across each chronic illness agreed that health apps have the ability to dramatically improve health. Although it is promising that one-third of people with chronic conditions report belief in health app efficacy, it remains that only a minority of at-risk populations would be likely to use health apps to improve their conditions and that most either do not know they exist or believe that apps could be helpful. Interest in and use of these apps will likely remain low and that motivating download of these resources among high-risk populations remains a critical challenge for the field.

Among research on health apps with chronically ill populations, another area of emphasis has been designing apps tailored to chronically ill patients. For instance, research has developed apps for assisting with specific disease management functions, such as improving medication adherence [16,18], and also for promoting healthy lifestyle choices among these populations [18]. According to our findings, although slight variations between conditions were identified, the most common reasons for health app download among individuals with chronic illness had to do with healthy lifestyle behaviors, such as tracking exercise, improving nutrition, and assisting with weight loss. The nuance in responses between conditions could in part be explained by different treatments for each condition. For instance, just over one-third of individuals with obesity reported most use of health apps for exercise tracking, as this is consistent with treatment for their condition, yet depression management would not necessarily require regular tracking; thus, less than one-third reported use of health apps for this function. However, hypertension and cholesterol are conditions that are largely dependent on exercise and nutrition but fewer reported use of health apps for these functions, suggesting differences in characteristics of the apps they are using or less adherence in modifying these behaviors. It is interesting to note that these were also the most common uses of health apps among populations without chronic illness.

Our results meaningfully extend the literature on health apps in several ways. Interestingly, our results found no significant difference in likelihood of health app download between individuals with and without chronic illness. That is, individuals with health apps were not more likely to have chronic health conditions than those without health apps. This could be due to the fact that use of health apps reported by participants in this study was actually quite high among those with and without chronic illness. In addition, individuals with chronic illness represented less than half of our sample. Nevertheless, we found individuals with *very good* and *excellent* self-reported health to be more likely to report health app download than individuals with *poor* self-reported health. We also found individuals with any report of regular physical activity (from *1 day per week* to *5-7 days per week*) to be more likely to report health app download than individuals without physical activity habits.

Our study extends the literature and our understanding of health app use and beliefs about these tools by comparing responses from individuals with markers of good health and those with markers of poor general health. Taken together, our findings suggest preliminary evidence that individuals who are using health apps may be those already engaging in healthy lifestyle behaviors. There may be an opportunity to better market health apps toward chronically ill populations, or design tailored apps specifically for these groups.

### Limitations

Despite strengths, this study was not without limitations. The primary limitation is the cross-sectional survey. In addition, our sample was skewed toward younger populations, and a more generalized sample across age would likely have yielded different results as patterns of use and preference are likely to be different in older populations. One example of sampling bias is the low prevalence of participants with a history of cancer (<5%), whereas the lifetime risk of developing cancer is about 40% for men and women. Sampling a more diverse or broad sample may have achieved different findings. It should also be noted that individuals without chronic illness represented a large portion of the sample (n=807). Additionally, the study surveyed a general population rather than those known to be medically ill. Hospital- or clinic-based populations that regularly receive health care may differ in their behaviors and current uses of health apps. Furthermore, the groups of comorbidity were heterogeneous, presenting a limitation in the ability of the findings identified here to apply to all individuals with comorbidity. Opinions of health app use could change over time, and although we used a validated instrument to assess chronic medical conditions, it nevertheless relied on self-reported data. Furthermore, the potential uses of mobile health and medical apps are nuanced and varied in nature. It would be challenging to capture the numerous and varied uses and types of apps. The results of this study are limited and may not capture all potential uses of apps or types of apps for health or disease management. It should be noted that the authors measured health app *download* and app use *frequency* in the survey. The authors chose the terminology *download* to align closely with the actual behavior being conducted (downloading a health app) and then assessed frequency to understand how often respondents engage with health apps.

### Future Research

Our study extends the literature and our understanding of health app use and beliefs about these tools by comparing responses from individuals with markers of good health and those with markers of poor general health. Our findings illuminate not only behavioral patterns of healthy individuals but also those of individuals with poor general health indicators (eg, low self-rated health). We also found lower download among individuals who may need these interventions the most. The results of this study suggest high use of health apps among individuals with high self-rated health and physical activity.

The study illuminates future research on public health interventions to promote mobile health uptake among individuals with chronic conditions. Future interventions may consider how best to tailor health apps toward individuals with specific conditions and the needs of those conditions (eg, weight management among individuals with obesity) or identify ways to better communicate health apps and their benefits to those with chronic illness. More trials and well-designed studies can help provide data regarding efficacy of specific health apps to change the cost-value perception among both patients with chronic conditions and health care providers. Furthermore, designing targeted interventions may be a strategy for easing the burden of complex treatment regimens and promoting health in populations with chronic conditions.

### Conclusions

Mobile technology is increasingly low cost and well suited for population health. Although there is interest in applying mobile technology to health and particularly to disease management, little attention has been paid to current use in populations with chronic health conditions. Our study found no difference in health app use between healthy and chronically ill populations, but we did find self-reported health and physical activity to be the strongest predictors of health app use. Our study also found approximately one-third of individuals with chronic illness reported beliefs that health apps have potential to improve health, suggesting these tools could be better marketed toward individuals with chronic illness. Results have direct application for health communication and intervention to promote population health and assist individuals with chronic disease management.

## Abbreviations

OR: odds ratio
